# How consumer group communication influences brand memory during product injury crises

**DOI:** 10.3389/fpsyg.2022.922643

**Published:** 2022-09-02

**Authors:** Lei Wang, Yuxin Wu, Yuming Wan

**Affiliations:** ^1^College of Economics and Management, Northeast Agricultural University, Harbin, China; ^2^Development Research Center of Modern Agriculture, Northeast Agricultural University, Harbin, China

**Keywords:** consumer group communication, consumer product image perception, brand memory, consumer group involvement, consumer

## Abstract

Drawing on the social exchange theory, this study adopted a cross-level framework to investigate the influence of consumer group communication on consumer product image perception and brand memory. In addition, this paper examined the moderating role of consumer group involvement in the cross-level relationship between consumer group communication and consumer product image perception. Based on a sample of 116 groups and 530 consumers, results revealed that consumer group communication has a significant positive influence on brand memory formation across levels. Consumer product image perception plays a cross-layer mediated role between consumer group communication and brand memory. Group involvement plays a cross-level negative moderating role between consumer group communication and consumer product image perception, and moderates the mediating role of consumer product image perception between consumer group communication and consumer brand memory across different levels. Finally this paper discussed implications for research and practice.

## Introduction

In market competition, brand is an important intangible asset for enterprises (Yorkston et al., 2010) which represents consumers’ overall image of the enterprise (Feng, 2015). Brand is both an important bridge connecting consumers and the market and the core driving force for the survival and development of enterprises. Establishing a benign relationship between brands and consumers plays helps enterprises create solid positions in competitive market environments. However, with the continuous development of social economy, the competition among enterprises is increasingly fierce, and many enterprises ignore product quality and safety risks to seize the market, resulting in product injury crisis events. In addition, consumers’ convenient access to information creates a much wider negative impact from product harm events, and can even directly affect brand loyalty and brand performance (Ma et al., 2010). This can cause a sharp drop in sales and market share, leading to bankruptcy, which can also spread to the operation and development of other industries or enterprises. Even if a quality inspection report is issued by an authoritative department, consumers’ negative impression of the brand in crisis may remain. Influenced by the Country of Origin image and perception of quality (Islam and Hussain, 2022; Saeed et al., 2022), he first choice of consumers is still to buy imported products or find someone to help them purchase foreign products (Wang et al., 2020).

Otherwise, the product injury crisis events destroy the brand memory of consumers, and repeated crisis events will continue to deepen the destruction of consumer brand memory. Therefore, during a product injury crisis, one primary strategy for enterprises to get out of their predicament is by understanding the factors which influence the formation of consumer brand memory in order to reshape it.

In general, consumers lack trust in industries, enterprises, and products that have experienced crisis events. This is reflected in consumers’ purchase process of consumers, and leads to a generally low brand reputation and consumer purchase intention (Zhou et al., 2021). Therefore, consumers will be more cautious in purchasing decisions, be more inclined to seek others’ opinions and help, choose to communicate with other consumers about their purchasing experience (Yin and Poon, 2016), and continue to communicate and interact with their consumer groups after purchasing (Altinay et al., 2018). Even when consumers have a negative post-purchase evaluation, their original attitude will be changed through communication between groups, thus affecting their purchase behavior (Ding et al., 2021). Studies have shown that consumer-to-consumer interactions can both increase consumers’ brand knowledge and enhance consumers’ trust in brands and their products (Srivastava and Kaul, 2014). Research also shows that consumers’ brand memory is the basis of brand cognition, and therefore the first step toward consumers making purchases (Wang et al., 2020). When the group communication between consumers helps them gather new product information about brand, their original cognition and attitude will change, and the new brand memory will affect the purchase decision behavior. However, existing studies have not explored the relationship between consumer group communication and brand memory. This study aims to explore that relationship.

Communication between consumers is based on the purchase of the same brand or category of products. If communication occurs between two or more people, it will generally encourage other consumers to respond. Under normal circumstances, the interaction between individuals and groups generally includes product information collection and interpersonal communication (Khler et al., 2011). When consumers communicate, an interactive relationship group of more than one person is formed. According to social exchange theory, the interaction between people in a society is an exchange process, which is dominated by rationality and tends to have the largest benefits and the smallest costs (Cook and Whitmeyer, 1992). This exchange behavior begins with social attraction brought by common interests. For consumers, common interest among consumers is mostly reflected in the exchange of their own consumption experience, experience with a brand product (Zhao et al., 2016), and word of mouth recommendation (Sun and Guo, 2016). Therefore, according to social exchange theory, it is reasonable to think that information obtained through consumer groups is more trustworthy to consumers, and will therefore affect the cognition and attitude of individuals toward information along with the processing of information by consumers. At the same time, existing studies have shown that the product information (Dorsey et al., 2016) and brand knowledge (Srivastava and Kaul, 2014) obtained by consumers through communication will not only change the individual’s attitude and decision-making behavior (Patnaik et al., 2013), but also affect the individual’s perceived functional value, which then affects the purchase intention (Gremler and Gwinner, 2000). Consumer group communication will affect some individual consumer’s views and perceptions toward the brand or product across different levels, and then change their views and attitudes toward the brand. This study therefore aims to explore the cross-level mediating role of consumer product image perception between consumer group communication and consumer brand memory.

Both individuals and groups of consumers are part of the consumer market environment, so they are easily affected by the market environment, which then affects consumer psychology and consumer behavior during product injury crisis events and other situations. A product injury crisis refers to an event where a product is defective or dangerous to consumers, and this is widely publicized (Siomkos and Kurzbard, 1994). During product injury crisis events, consumers have anger and resistance to the brands or products involved. Scholars have examined the negative emotions generated by crisis events relating to their degree of involvement in negative publicity. Trump’s (2014) research shows that consumers who are more closely related to the product may react negatively when they receive negative information about the brand or the brand violates an ethical code. Specifically, consumers have a high level of involvement, they spend more time and effort to understand the brand and product. However, when their degree of involvement is low, they will spend less time and energy and will make a quick purchase decision (Lyu et al., 2018). Different degrees of involvement create different consumer understandings of the product. Therefore, in group communication, product information sharing will change due to different involvement levels, and consumers’ perception of product image will also change accordingly. This study aims to explore the cross-level regulatory role of consumer group involvement between group communication and product image perception.

At the same time, with the change in product image perception, brand memory will also change. Specifically, the communication atmosphere formed within the group differs with the involvement degree of different groups; as the perception of product image generated by information analysis and extraction of individual consumers also differs, the impact on brand memory also changes. This study explores the cross-level mediated effect of consumer group involvement on product image perception. To summarize, this paper takes product image perception as the mediating variable and group involvement as the moderating variable to build a moderated mediating model to study the mechanism of consumer group communication on brand memory.

## Theoretical background and hypothesis development

### Consumer group communication and brand memory

Consumer group communication refers to the process of information transfer from one individual or group to another individual or group, mainly focusing on various forms of verbal or non-verbal information transmission (Martin and Pranter, 1989). Brand memory refers to consumers’ stored and accumulated memories of brands and products in terms of effect, emotion, value and symbolism under different situations (Wang et al., 2020). When a harm crisis occurs, consumers get large amounts of information from enterprises, public opinion media, and other consumers, which will affect the attitude and purchase intention toward products involved in the crisis.

Based on the social exchange theory, social exchange is the foundation of social life, and people will examine the behavior of both parties at the cost of rewards and expenses (Homans, 1958). As a valuable way of interaction, group communication is a social exchange behavior. When the two parties in the communication were shared information about the brand, they will get the greatest return at the least cost. But people do not like being used or taking advantage of others. For this reason, group communication will abide by the principle of mutual benefit (Shin et al., 2006). People will get satisfaction from their fair social relations, and trust will establish in many successful communication and exchanges.

According to social exchange theory, consumers tend to listen to and internalize the opinions of trusted parties. As Penley and Hawkins (1985) believed, the basic problem of “what to communicate,” that is, the content of communication, must be involved in communication. According to the characteristics of rational consideration and frequent interpersonal interaction in dealing with things in Chinese society (Ji, 2008), compared with enterprises and public opinion media, consumers communicate more frequently, so it is easier to obtain information about brands and products immediately, generate group identity, listen to suggestions and opinions within the group, and create an impression. This promotes the generation of consumers’ brand memory. Other studies have shown that consumer attitudes will ferment and amplify under group interaction, and then group members will have the same view, so group communication will affect group member behaviors (Xu and Su, 2015). Therefore, it can be speculated that consumer group communication will have an impact on consumer brand memory. Based on the above analysis, the following assumption is proposed:

*Hypothesis 1*: Consumer group communication has significant positive influence on brand memory formation across levels.

### The mediating role of consumer product image perception

Product is the concrete manifestation of brand. As a concrete thing, it forms a specific image through our perception system. As a dimension of brand image, product image is consumers’ perception of product related information after understanding brand products (Keller, 1993). Therefore, “consumer product image” in this paper refers to the specific image left in the mind of consumers through their overall understanding of the product, including the internal and external characteristics of packaging, price, quality, safety, and word-of-mouth. Along with their own purchase experience, consumers’ access to product information depends on communication with other consumers. Shen et al. (2016) believes that customer interaction will reduce customers’ uncertainty about products, affect their attitudes, and then affect their purchase behavior. According to the social exchange theory, consumer group communication will enable consumers to share the brand and product information they know, which includes not only the objective information about the brand and product, but also their understanding and attitude toward the brand (Zhao et al., 2016). Communication will deepen consumers’ understanding and trust with each other, which affects whether consumers trust the information they share and the perceived effect (Gremler and Gwinner, 2000). In communication, the speed of information feedback among consumer groups also affects consumers’ trust in information sharing within their groups to a certain extent (Sun and Guo, 2016). When the information provided by the consumer group has a certain purchasing guidance effect on the consumer, the consumer will deepen the impression of the product. According to the social exchange theory, when members have high-quality social exchanges, it is conducive to the formation of positive interactions between the two parties. When consumers’ product image perception is formed, consumers will also give back to their group members as a social exchange, improve consumers’ trust in the group, and form a virtuous circle. Therefore, it can be inferred that under the effect of group communication, consumers will have an impact on the image perception of brands and products. Based on the above analysis, the following assumption is proposed:

*Hypothesis 2*: Consumer group communication has a significant positive impact on consumer product image perception at different levels.

According to the associative network memory model, when consumers obtain information from the outside, an information node in their brain will be activated. When the information node spreads to its associated nodes and reaches a critical value, the information node will be recalled (Keller, 1993). Therefore, Keller (1993) believes that in the brand knowledge map, the brand image is an information node that arouses consumers’ memory, and when these information nodes are activated, the consumers will recall information about the brand or product, which promotes their purchase behavior. Studies show that brand cognition, brand name, quality, packaging and advertising are the main factors affecting brand memory (Joshua and Hurwitz., 2009; Jennifer et al., 2015). Among these factors, consumers’ perception of product image is more intuitive, easier to share and communicate, and has a more lasting memory, which plays an important role in the formation of consumers’ brand memory. When consumers mention or buy a brand’s production again, the reactivated product image perception will make consumers recall the memory of the brand, and choose whether to buy or not according to the memory. According to social exchange theory, when consumers can recall more positive perceptions, they will have more trust in the brand, and the better the brand memory effect, the more likely they are to engage in consumption behavior. At the same time, when consumers have more brand memory of a certain brand, they will be more inclined to this brand in the selection of similar products because of trust. Based on the above analysis, the following assumption is proposed:

*Hypothesis 3*: Consumers’ perception of product image has a significant positive impact on the formation of brand memory.

To sum up, consumer group communication affects the brand and product itself and the emotional communication of consumers within the group, which will affect the product image perception of the brand and products among group members; the consumer product image perception promotes consumers’ cognition, understanding, and attitude toward brand products, and effectively promotes the formation of consumers’ brand memory. According to the social exchange theory, the positive interaction established between consumer groups will enhance the level of trust between consumers. Therefore, group communication will enable consumers to have a more comprehensive understanding of a brand and its products, and will also collect more information about products. Help consumers establish product image perception from multiple perspectives, and then form corresponding brand memory to help consumers make judgments. Good group communication will deepen consumer product image perception and enable consumers to form a more comprehensive brand memory. Based on the above analysis, the following assumption is proposed:

*Hypothesis 4*: consumer product image perception plays a cross-layer intermediary role between consumer group communication and brand memory.

### Cross-level moderating of group involvement degree

Consumers are in social groups. In addition to their own perception of brands and products and communication with consumers, the ways that both consumers and groups are involved with products will change how group communication affects product image perception (Chai, 2020). The degree of involvement is the correlation between oneself and an object perceived by individuals according to their own differentiated interests, needs, and values (Zaichkowsky, 1985).

For consumers, the involved object may be a product or brand, an advertisement or motivation. Zaichkowsky (1985) believes that there are three types of involvement: advertising, product, and purchase decision involvement. The last type of involvement refers to consumers’ attention to a shopping object or behavior. Specifically, if consumers perceive that products or purchase activities are more important to them, they will invest more time in product or purchase activities for information collection and comparison; this is considered a high degree of involvement (Zhang and Chen, 2008). However, if a certain consumption process perceived by consumers is not important to them, they are generally unwilling to invest too much energy in collecting information and comparison, which is a low degree of involvement (Chai, 2020). Consumers with high involvement have a better understanding of the information required for products or purchase activities than consumers with low involvement, have a higher sensitivity to product characteristics (Melvin, 2020), have their own views and attitudes toward products, and are less affected by interference from external factors. When the degree of involvement is low, consumers are more likely to accept all kinds of external information because they pay less attention to product information, meaning they will not invest a lot of time and energy into understanding and selecting a product. Therefore, in groups, the effect of product image perception through group communication will also vary with the degree of involvement. This paper examines the degree of group involvement as it refers to the overall situation of all consumers of a product. Therefore, for consumer groups with low group involvement, it is easier for group communication to promote the formation of consumer product image perception. Consumer groups with high group involvement struggle more to use group communication to promote the formation of consumer product image perception. Based on the above analysis, the following assumption is proposed:

*Hypothesis 5*: Group involvement plays a cross-level negative moderating role between consumer group communication and consumer product image perception.

### Mediations that are mediated across levels

Group communication contributes to the formation of consumers’ perception of product image, but whether consumers can transform the perceived product image into brand memory also depends on the degree of consumer group involvement. This is mainly because the formation of memory is relatively complex and stable. Consumers’ willingness to collect information varies with their degree of involvement (Rokon et al., 2020), which leads to a deviation in consumers’ internalized memory of product image perception after group communication. Specifically, for consumer groups with low group involvement, information shared within the group will directly affect consumers’ perception of product image and thus form brand memory. In addition, the low involvement group will not extensively search for or consider the accuracy of information obtained from group communication. When information sharing within the group stimulates consumers, consumers will make direct judgments about products and brands and form memories. This also explains why, during a product injury crisis, some consumers will continue to hold negative brand memories no matter how many positive reassurances authorities give. In those with a high level of group involvement, however, although the group may have a good atmosphere for product and brand information sharing, members will continue to collect relevant information after they become interested to confirm the accuracy of the obtained information. Consumers’ perception of product image is formed more directly from the information obtained through communication among consumer groups, which weakens the impact of product image perception on brand memory. Based on the above analysis, the following assumption is proposed:

*Hypothesis 6*: Group involvement significantly negatively moderates the mediating role of consumer product image perception between consumer group communication and consumer brand memory across different levels. The lower the group involvement, the stronger the effect of group communication on consumers’ brand memory through consumers’ product image perception, and vice versa.

**Figure 1:**
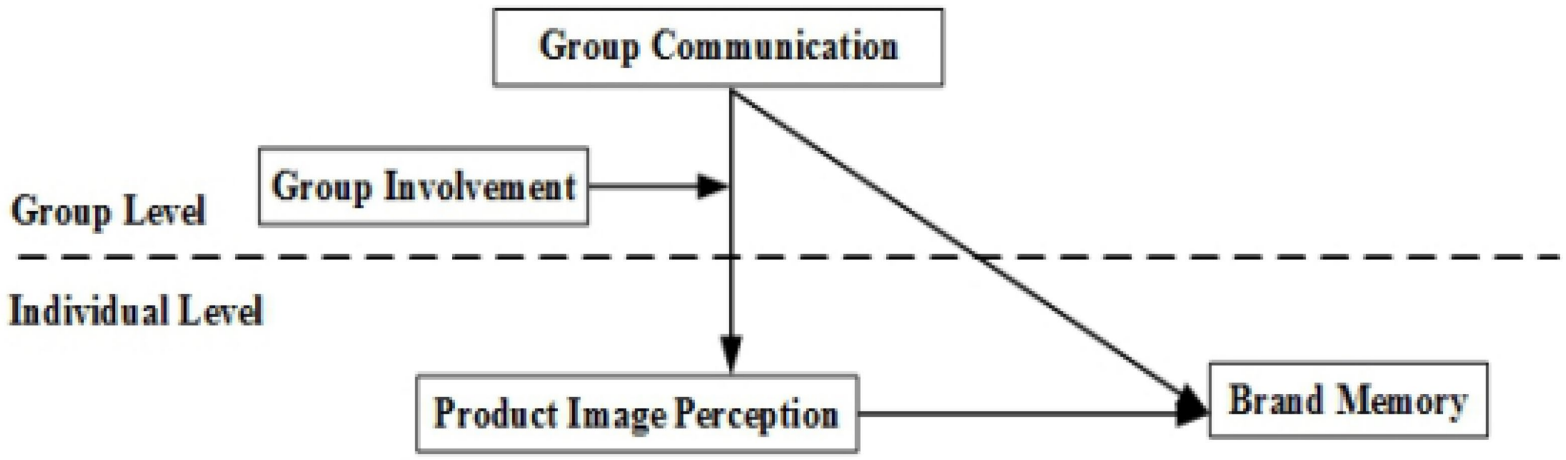
Conceptual model of how consumer group communication influences consumer brand memory.

Based on the above discussion, a cross-level theoretical model of the impact of group communication on consumers’ brand memory is constructed (see Figure 1).

## Materials and methods

### Sample and procedures

In order to study more generality, our research covers different work backgrounds and different types of consumers as much as possible when selecting samples. The sample is selected from consumers who have purchased dairy products. The specific criteria are as follows: (1) In China’s product injury crisis events, dairy injury crisis events occur frequently and have a great impact on consumers (Wang and Li, 2016). Therefore, research samples were selected with this background in mind. First, we should confirm whether the sample is a dairy consumer, defined as whether they have purchased one or more dairy products. (2) The number of people in the group needs to be three or more. (3) The frequency of group communication should be more than five times a week. (4) Sample occupations should be as diverse as possible.

Before the official distribution of the questionnaire, first, the researcher got in touch with the target group and the relevant responsible persons in the group and explained the content and process of this investigation in detail. After obtaining the consent of the person in charge, the number of subjects who voluntarily participated in this survey was obtained, and the time and number of subjects were determined to issue questionnaires on site. Questionnaires of the individual affiliation group and individual are mapped one-to-one through implicit numbers, the subjects are informed in advance of the anonymity and confidentiality of the questionnaires, and the survey results are only used for academic research and other matters needing attention. To ensure data security after completing the questionnaires, the questionnaires were collected and sealed on site. For researchers who are unable to distribute the questionnaires in person, the questionnaires will be distributed online. To ensure the anonymity and confidentiality of the questionnaire, the content, statistical analysis, and detailed information of the questionnaire are set to be private so that the subjects can fill it out with confidence.

This study’s field survey questionnaire primarily comes from the consumer groups in the three northeastern provinces. Major supermarket brand enterprises, such as Walmart, Yonghui, Carrefour, and other voluntary consumers, are mainly selected, and they are required to help researchers to provide group and member information. The researcher screened qualified consumer groups according to the group selection criteria and distributed questionnaires after obtaining the consent of the members of the group. Before the questionnaire was distributed, participants were told to fill in the questionnaire anonymously, and the results could only be viewed by researchers, and the data obtained were only used for academic research. Questionnaires were distributed with the consent of the participants. The online survey mainly selects social software such as WeChat group and QQ group that meet the group selection criteria, using anonymous questionnaire star software to fill in, choose a questionnaire in a different time, to provide exclusive within the group number (e.g., date + letters), to ensure the sample completed within the same group and then on to the next group to fill in, after complete set system random red envelopes, coupons, etc. as thanks.

A total of 600 questionnaires were distributed in this study, which was distributed at multiple time points. The individual questionnaire includes scales such as consumer basic information, product image perception, and brand memory, and the group questionnaire includes scales of group basic information, group communication, and group involvement. Individual questionnaires are distributed and returned 24 weeks after the group questionnaire is collected. Excluding the questionnaires that could not be paired, had too many multiple choices, too many omissions, obvious response tendencies, or did not meet the above collection principles, 530 valid questionnaires filled in by 116 groups of consumers were finally obtained, and the effective recovery rate was 88.3%. Specific information is shown in Table 1.

**Table 1:** Basic situation of the sample.

Title	Options	Number	Percentage
Gender	Men	165	31.10%
	Women	365	68.90%
Education Level	Doctor’s degree	6	1.10%
	Master’s degrees	78	14.70%
	Undergraduates	391	73.80%
	Below undergraduate	55	10.40%
Age	Under the age of 18	35	6.60%
	19–30	397	74.90%
	31–45	73	13.80%
	45–60	22	4.20%
	over 60	3	0.60%
Occupation	Students	310	58.50%
	Company employees	81	15.30%
	Self-employed	49	9.20%
	Teachers	17	3.20%
	Civil servants	13	2.50%
	Senior managers	8	1.50%
	Other occupations or inconvenient disclosure	52	9.80%
Group Size	3–4 persons	65	56.04%
	5–6 persons	40	34.48%
	7–8 persons	10	8.62%
	more than 8 persons	1	0.86%
Group Communication Frequency	5–7 times a week	25	21.55%
	8–10 times a week	9	7.76%
	11–13 times a week	26	22.41%
	more than 13 times a week	56	48.28%

### Measures

The measures of all constructs were adapted from previous researches. A Likert-type scale was used to assess the extent to which the respondents agreed with the 35 items in the questionnaire, where 1 = strongly disagree and 5 = strongly agree. The specific scale is as follows.

Group communication mainly refers to the scale compiled by Penley and Hawkins (1985) and modified in combination with the specific contents of this study, with a total of 13 items. In this study, the reliability coefficient of the scale is 0.894. Items include “when referring to a certain brand of dairy products, your colleagues, roommates or friends will give advice to each other,” “when I ask questions, our colleagues, roommates or friends always try their best to give an answer,” “our colleagues, roommates or friends will recommend dairy products to each other,” etc.

Product image perception mainly refers to the scale compiled by Biel (1992) and modified in combination with the specific contents of this study, with a total of 6 items. Items include “you think the price of dairy brand products you currently buy is very affordable,” etc. In this study, the reliability coefficient of the scale is 0.768.

Brand memory mainly refers to the scale compiled by Wang et al. (2020), with 12 items in total. In this study, the reliability coefficient of the scale is 0.913. Items include “I will generate positive views on a dairy product brand through the recommendation of my family, colleagues and friends,” “I think the dairy product brand can attract the attention and respect of others to some extent,” “if a dairy product brand promises a quality product and will assume social responsibility, I will buy the product with confidence,” “When I buy products from this brand, I will pay attention to the consumption experience they provide,” etc.

The degree of group involvement mainly refers to the scale compiled by Zaichkowsky (1985) and modified in combination with the specific contents of this study, with a total of 4 items. In this study, the reliability coefficient of the scale is 0.869. Items include “dairy products are important to me,” etc.

From the perspective of individual consumers, because the demographic characteristics of each consumer are different, their gender, age, education and occupation may affect consumers’ brand memory. From the perspective of consumer groups, since the samples of this study come from different consumer groups, the communication frequency and group size within the group will also affect consumer group communication and brand memory. Therefore, this study takes the gender, age, education level, and occupation of consumers as the control variables at the individual level, and the group communication frequency and group size as the control variables at the group level.

### Data analysis methods

SPSS 25.0, Amos 23.0, Mplus 7.4 and R language programs were used for data analysis. (1) Using SPSS 25.0 and AMOS 23.0 software, the discriminant validity of the scale was tested by confirmative factor analysis. (2) SPSS 25.0 software was used for descriptive statistical analysis and correlation test between variables. (3) By calculating the data aggregation index, SPSS 25.0 software is used to determine whether the individual level data is suitable for aggregation to the team level. (4) Using Mplus 7.4 software, according to the theoretical model studied, the cross-level analysis method is used to test the theoretical hypothesis of this study to test the significance of cross-level mediating effect and moderating effect, because this method has more advantages in dealing with non-normal data. (5) The indirect effect of the adjusted cross-layer intermediary effect is tested by using the R language program. The 95% deviation corrected confidence interval is obtained by the bootstrapping method (repeated sampling 5,000 times) to test the significance of the regression coefficient. If the confidence interval (CI) does not contain zero, the corresponding effect is significant.

## Results

### Data analysis methods

Before distinguishing and confirming the convergent validity and discriminant validity of each variable, Harman single factor test showed that the variance explained by the first factor was 34.646%, less than 40% of the critical standard, indicating that there was no serious common method deviation in this study (Podsakoff et al., 2016). Therefore, factor analysis was used to test the structural validity of the scale. KMO = 0.929, Bartlett = 13201.667, which is significant at the level of *p* < 0.001, so factor analysis can be carried out. Then, the results of confirmatory factor analysis on group communication (represented by X), product image perception (represented by M) and brand memory (represented by Y) are shown in Table 2. Table 2 shows that the fitting index of the three-factor model is the best and far better than other competitive models. However, although this study uses a multi-level and multi-source data collection method, the answers to each variable are from the individual level. There may still be common method deviation problems. Therefore, based on the three-factor model, We adds a common method deviation factor to construct a four-factor model. The results show that the four-factor fitting index is =2.813、RMSEA = 0.059、IFI = 0.931、TLI = 0.918、CFI = 0.931. Comparing the results of the three-factor model and the four-factor model, △RMSEA = 0.001, the difference between the two results is not more than 0.05, △IFI = 0.001、△TLI = 0、△CFI = 0.001, the difference between the two results is not more than 0.1. The four-factor fitting index is not significantly better than the three-factor model, indicating that there is no serious common method deviation problem in this study (Liu et al., 2015).

**Table 2:** Confirmatory factor analysis results.

Model		df		*RMSEA*	*IFI*	*TLI*	*CFI*
Three - factor model (X、M and Y)	1118.097	398	2.809	0.058	0.930	0.918	0.930
Two - factor model (X + M and Y)	2383.005	422	5.647	0.094	0.809	0.789	0.809
Two - factor model (X and M + Y)	3203.211	426	7.519	0.111	0.730	0.704	0.729
Two - factor model (X + Y and M)	3833.146	433	8.853	0.122	0.669	0.644	0.668
Single factor model (X + M + Y)	4489.008	434	10.343	0.133	0.605	0.576	0.604

X represents group communication. M represents product image perception. Y represents brand memory. The same below.

### Descriptive statistical analysis of variables

Table 3 shows the descriptive and correlation among group communication, product image perception, group involvement, and brand memory. Among them, the correlation coefficients between group communication and product image perception and brand memory have reached 0.615 (*p* < 0.01) and 0.774 (*p* < 0.01), indicating that group communication is closely related to product image perception and brand memory, which is in line with the theoretical expectation.

**Table 3:** Descriptive and correlation analysis of main variables (*n* = 530).

Variable		Mean	Standard deviation	1	2	3	4	5	6	7	8	9
1	sex	1.69	0.463									
2	age	2.17	0.626	−0.154^**^								
3	edu	2.07	0.541	0.060	−0.151^**^							
4	job	2.46	2.066	−0.090^*^	0.356^**^	−0.183^**^						
5	GCF	3.92	1.236	−0.017	0.032	0.028	−0.103^*^					
6	GS	2.73	0.759	−0.096^*^	−0.013	0.044	0.147^**^	−0.184^**^				
7	X	4.095	0.496	0.078	0.138^**^	−0.091^*^	−0.059	0.230^**^	−0.153^**^			
8	M	4.058	0.615	0.130^**^	0.056	−0.110^*^	−0.023	0.150^**^	−0.056	0.615^**^		
9	Y	3.960	0.615	0.012	0.159^**^	−0.098^*^	−0.026	0.278^**^	−0.096^*^	0.774^**^	0.604^**^	
10	W	3.877	1.107	−0.019	0.055	−0.033	0.101^*^	0.087^*^	0.011	0.055	0.067	−0.002

* *p* < 0.05;

** *p* < 0.01.

the same below. GCF represents group communication frequency. GS represents group size. W represents group involvement.

### Data aggregation

Group communication and group involvement are variables at the group level. Therefore, we first need to check the consistency of opinions among members within the team to aggregate the individual level data to the group level. This paper uses the intra group rater reliability Rwg(j) and intra group correlation coefficient ICC to judge whether the perceived degree of group communication and group involvement at the individual level can converge to the group level. The results showed that ICC(1) = 0.758 and ICC(2) = 0.935 were higher than the critical value of 0.500. The average Rwg(j) is 0.985, which is greater than the critical value of 0.700 (James et al., 1984). ICC(1) = 0.778 and ICC(2) = 0.941, which were higher than the critical value of 0.500. The average Rwg(j) is 0.823, which is greater than the critical value of 0.700 (James et al., 1984). Therefore, it is appropriate and effective to aggregate the group communication and group involvement perceived from the individual level to the group level.

### Hypothesis test

Since the data used in this study include both the group and the individual level, group communication and group involvement are at the group level, and product image perception and brand memory are at the individual level, considering that the samples in the same group are not independent individuals to a certain extent. Therefore, the path of group communication affecting brand memory through product image perception involves a cross-level role. In this study, the cross-level analysis model is adopted, and the zero model is tested first to test the rationality of cross-level analysis.

#### Zero model test

Zero models with product image perception and brand memory as outcome variables were set to investigate the intra group and inter group variance. The results showed that the intra group variance and inter group variance of product image perception were 0.374 and 0.147 respectively, ICC(1) = 0.502, ICC(2) = 0.822. The intra group variance and inter group variance of brand memory were 0.096 and 0.361 respectively, ICC(1) = 0.758, ICC(2) = 0.935. The results showed that the values of ICC(1) and ICC(2) of product image perception and brand memory exceeded the critical value of 0.500. Therefore, the variation based on product image perception and brand memory can be explained by group level variables and multi-layer linear regression analysis can be carried out.

#### Main effect test

After controlling the gender, age, education level, occupation, group communication frequency and group size of consumers, group communication at the group level has an impact on brand memory (M5, *β* = 1.150, *p* < 0.001), had a significant positive effect, and the results are shown in Table 4. Thus, hypothesis 1 is supported.

**Table 4:** Cross-level model test results (*n* = 530).

Variables/Model	M			Y				
	M1	M2	M3	M4	M5	M6	M7	M8
Intercept item	4.066^***^	4.059^***^	3.539^***^	3.963^***^	3.952^***^	3.975^***^	2.435^***^	1.191^***^
Individual level								
sex	0.060	0.072	0.063	−0.012	−0.018	−0.016	−0.027	−0.035
age	0.051	0.040	0.002	0.022	0.021	0.017	0.017	0.027
edu	−0.104^*^	−0.084	−0.060	−0.038	−0.028	−0.027	−0.019	−0.015
title	−0.019	−0.017	−0.002	−0.005	−0.005	−0.003	−0.003	−0.002
M						0.116^**^	0.105^*^	0.107^*^
Group level								
GCF	0.073	0.010	0.005	0.110	0.030	0.108^*^	0.028	0.033
GS	−0.040	0.043	0.051	−0.063	0.059	−0.063	0.047	0.043
X		0.820^***^	0.604^***^		1.150^***^		0.837^***^	0.670^**^
W			0.097^**^					−0.099^**^
X × W			−0.274^***^					0.184^*^
Variance decomposition								
σ^2^	0.188^***^	0.189^***^	0.189^***^	0.063^***^	0.063^***^	0.061^***^	0.061^***^	0.061^***^
Τ_00_	0.194^**^	0.053^**^	0.023^*^	0.333^***^	0.050^***^	0.323^***^	0.043^***^	0.035^**^

*p < 0.05; **p < 0.01; ***p < 0.001.

#### Mediation test

According to Hypothesis 4, product image perception plays a positive mediating role in the relationship between group communication and brand memory. In this intermediary relationship, because only independent variables are defined at the group level, and intermediary variables and outcome variables are defined at the individual level, it belongs to the “2–1-1” intermediary model. According to the inspection procedure proposed by Baron and Kenny (1986), the “2–1-1” cross-level mediation model is divided into four steps. The analysis results are shown in Table 4.

(1) Step 1 and 2, respectively, test the zero model of brand memory and the main effect of group communication on brand memory, which has been supported. (2) In step 3, the impact of group communication on product image perception was tested. It was found that group communication (M2, *β* = 0.820, *p* < 0.001) had a significant positive impact on product image perception. Thus, hypothesis 2 is supported. (3) In Step 4, the product image perception was put into the equation, and the result showed that the product image perception (M6, *β* = 0.116, *p* < 0.01) had a significant effect on brand memory, and hypothesis 3 was supported. Then group communication and product image perception were put into the equation at the same time. The results showed that product image perception still had a significant impact on brand memory, and group communication (M7, *β* = 0.837, *p* < 0.001) had a significant effect on brand memory. To further verify the mediating effect of product image perception, this study uses the Monte Carlo method to repeat random sampling 20,000 times to calculate the confidence interval of mediating effect. The results show that the indirect effect of group communication on brand memory through product image perception is 0.312, and the 95% confidence interval is [0.03, 0.61], excluding 0. According to the above results, product image perception plays a mediating role between group communication and brand memory. Therefore, hypothesis 4 is supported.

#### Moderated mediating effect test

This step tested the moderating effect of group involvement. Table 4 showed that group communication had a significant positive effect on product image perception (M2). Group communication and group involvement had significant effects on product image perception, and the regression coefficients were 0.604 (M3, *β* = 0.604, *p* < 0.001) and 0.097 (M3, *β* = 0.097, *p* < 0.01), respectively. After the addition of the interaction term, the interaction term had a significant effect on product image perception (*β* = −0.274, *p* < 0.001), which indicates that group involvement plays a moderating role between group communication and product image perception. Hypothesis 5 can be verified.

**Figure 2:**
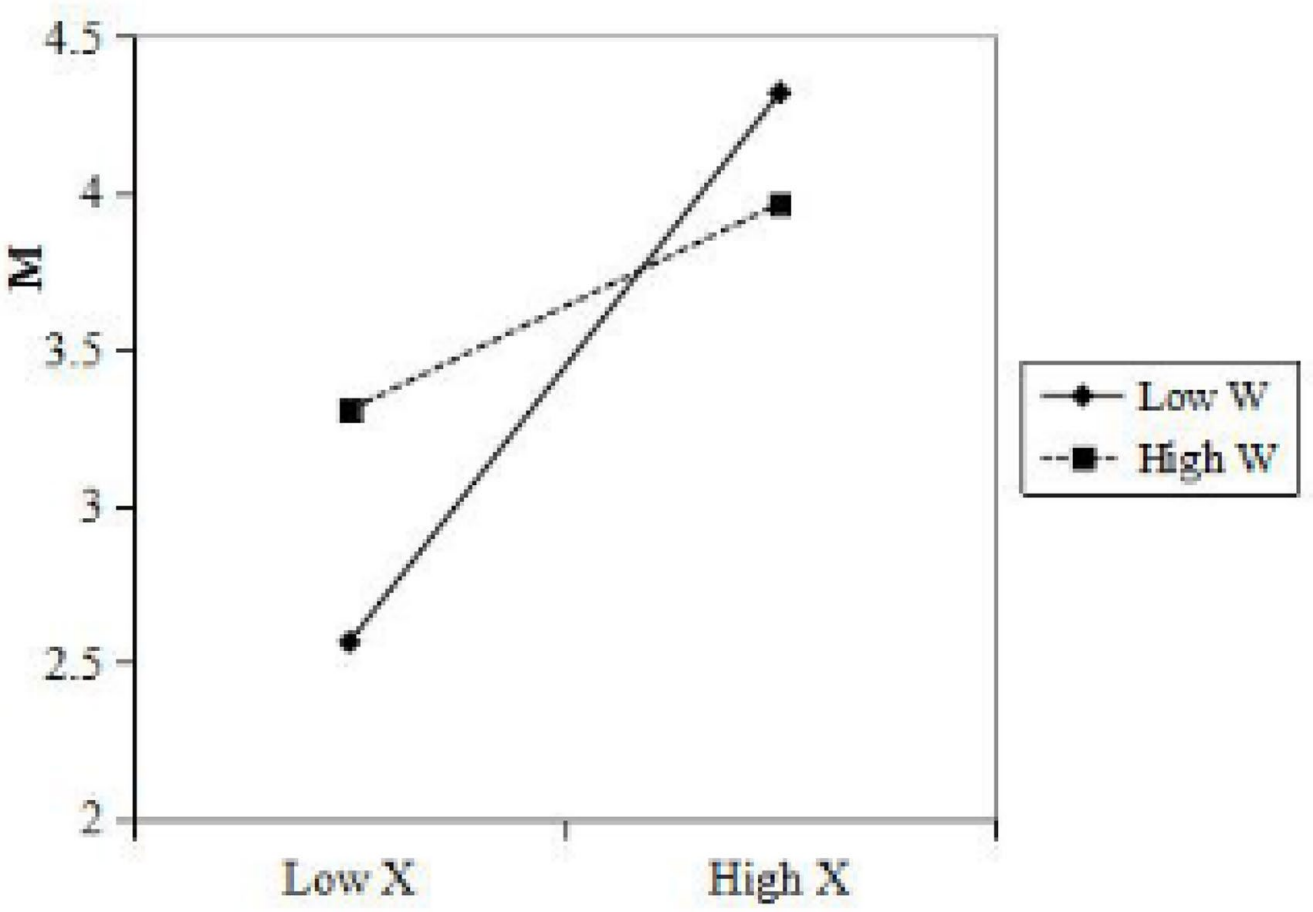
The moderating effect of group involvement on group communication and product image perception.

To more intuitively reflect the moderated effect of group involvement, this paper draws the regulatory effect interaction diagrams of group involvement at the level of one standard deviation higher and lower than the mean (as shown in Figure 2). The results of simple slope analysis showed that the degree of group involvement was higher (*β* = 0.330, *p* < 0.01), the relationship between group communication and product image perception under the condition of low group involvement (*β* = 0.878, *p* < 0.001), hypothesis 5 was further verified.

Hypothesis 6 was tested based on Preacher and Selig (2012) and the results are shown in Table 4. Group communication had a significant positive effect on brand memory (M5). After the addition of mediating variables and interaction terms, product image perception had a significant positive effect on brand memory (M8, *β* = 0.107, *p* < 0.05). After these steps, the effect of interaction term on brand memory was still significant (M8, *β* = 0.184, *p* < 0.05).

To further verify the indirect effects of cross-level mediated effects, according to Edwards and Lambert’s (2007) suggestion, this study used Mplus 7.4 to establish the path coefficient model, and added parameters on the basis of model 8, namely, high level of population involvement (+1SD) and low level of population involvement model (-1SD). Then, the R program was applied to perform 5,000 times of bootstrapping to obtain the confidence interval. The results are shown in Table 5. The results show that, in the case of high level of group involvement, the mediating effect of product image perception is significant, the coefficient is 0.481, 95% confidence interval is [0.051, 0.78], excluding 0. In the case of low group involvement level, the mediating effect of product image perception is also significant, with a coefficient of 0.729, 95% confidence interval of [0.087, 1.046], excluding 0. These results indicate that group involvement can moderate the mediating effect of group communication on brand memory through product image perception, so hypothesis 6 has been verified.

**Table 5:** Analysis results of cross-level moderated mediating effects.

Effect / Mediating path	Moderated variable	Coefficient	95% confidence interval	
			LLCI	ULCI
Indirect effect/ Product image perception	High group involvement	0.481^***^	0.051	0.78
	Group involvement	−0.278^***^	−0.363	−0.035	
	Low group involvement	0.729^***^	0.087	1.046

***p < 0.001.

## Conclusion

### Discussion and conclusion

In response to the calls for additional research from previous studies, this study investigated why and under what circumstances consumer group communication had an impact on the formation of consumer brand memory in the context of product injury crisis (Wang et al., 2020). By introducing product image perception from the perspective of consumer perception as a mediating variable and consumer group involvement as a moderating variable, the results show that consumer group communication has a significant cross-level positive impact on the formation of brand memory. According to social exchange theory, this study found that consumer product image perception is an important mechanism connecting consumer group communication and the formation of brand memory, which indicates that consumer group communication can affect consumers’ positive cognition and attitude about products, which can then affect consumers’ brand memory. The results also show that consumer group involvement can moderate consumer group communication across levels and influence consumer product image perception, which can then affect consumer brand memory. When group involvement is low, group communication can play a more significant role and promote consumers to form positive brand memories. This shows that in the context of the injury crisis, the reasons that can ultimately influence consumers to change their brand memory are related to consumers themselves and consumer groups. When consumers or groups know a lot about a product or brand, consumers will use group communication as a channel to collect information to supplement their cognition of the product or brand. At this time, consumers are often loyal customers of the brand. Once the brand memory is formed, it is difficult to change; when consumers themselves or the group do not know a certain brand, because they have less information and compare with members outside the group, consumers are more willing to trust members in the group to reduce risks. At this time, consumers’ cognition of the brand relies more on information sharing within the group, which leads to the content or results of group communication often making consumers directly remember the brand. This also further demonstrates the effectiveness of consumer group communication that is needed to take specific conditions (such as the degree of group involvement) as the premise and guarantee.

### Theoretical implications

First, previous studies had primarily focused on the impact of enterprise products and behaviors on consumer brand memory at the individual level (Feng et al., 2019). This study began from the novel perspective of group communication at the group level and verified that group communication has significant cross-level positive impact on consumer brand memory. Both expand the consumer group communication relationship with brand memory theory. Additional research should continue to explore other groups of consumers level variables (e.g., group identification, group interactions, group emotions, etc.) and consumer brand memory or other activities (such as buying decision behavior, brand communication, brand switching behavior intentions, etc.) to provide a useful reference.

Second, although existing research does focus on the mediating role of consumer perception perspective between consumers and brands, most studies look at the perception of products based on consumers’ personal characteristics, and few involve the impact of consumer groups on consumers’ personal perception. From the perspective of social exchange theory, this paper takes product image perception as a mediating variable at the individual level and tests the cross-level mediating effect of product image perception between group communication and brand memory. It can not only more completely present the complex transmission mechanism of group communication affecting brand memory, but it also effectively makes up for the deficiency in previous studies, providing an enlightening idea for subsequent studies on the mediating mechanism of consumer brand memory based on the group and individual levels.

Third, this study found that the degree of group involvement negatively moderated the effect of group communication on product image perception, and further moderated the indirect effect of group communication on brand memory through product image perception. Rooted in China’s local situation and product injury crises, it not only responds to the call of the academic community to examine China’s local situation, it also provides a local empirical argument for the significant impact of consumer group communication on consumers’ brand memory and cognitive behavior in the development of China’s domestic brand marketing.

### Managerial implications

First, enterprises can properly participate in the communication between consumers to improve consumers’ brand memory. For example, online sales channels can invite consumers who have purchased products to join product exchange groups to understand consumer needs and suggestions through communication with consumers. They can also be encouraged to recommend the product to friends or family members who are interested in the group and have the same purchase demand in exchange for secondary purchase discounts and gifts. This can help promote a good group communication effect.

Second, enterprises should create a good communication atmosphere between consumers and between consumers and enterprises, should also and provide necessary conditions and guarantees. For example, enterprises and brands can optimize product performance, services, and improve product quality according to consumer feedback. Enterprises can build online and offline communication platforms to provide hardware support for communication between consumers. By providing brand or product related knowledge, they can help consumers better understand the brand and product concept and improve the intention of positive communication among consumers.

Finally, enterprises should make special plans for groups with different levels of understanding. For example, when the group has a high understanding of the brand and product, it can provide more features and highlights of the product, as well as the core competitiveness and competitive advantages of its own product compared with similar brand products. When consumers have little to no information about brands and products, companies can provide a more intuitive level of product information, such as the high-quality raw materials, the preferential benefit price, exquisite packaging information, and similar items to stimulate interest from consumers and potential consumers. This will help them understand why the brand is interesting and create the foundation of a good brand memory, promoting purchase behavior.

### Limitations and directions for future research

Although this study tries to ensure the objectivity and scientificity of the research process, there are still some deficiencies.

Although the data sources are multi-time, multi-level and multi-reporter, it is still impossible to infer the potential causality. Future research can use combine data with experimental research to further explore the causal relationship between consumer group communication and brand memory.

This study mainly uses the questionnaire method to collect data, and the reporter makes a subjective evaluation of the items. Therefore, there is still a potential risk of common method deviation. To avoid this problem, future research can combine subjective evaluation with objective data, such as using objective indicators to evaluate consumer brand memory, to provide more effective data support.

This study verifies the restriction of involvement degree on the effectiveness of group communication. Future research can further explore other possible boundary conditions, such as group identity, to more systematically understand the factors influencing the formation of consumer brand memory.

In the process of data collection, this study also found that blockchain technology can provide a more secure and reliable data collection method, and provide technical guarantees of security, authenticity, and privacy for group communication effects and consumer behavior data. Therefore, in future research, it is possible to introduce blockchain technology to analyze consumers’ consumption behavior in the context of past consumption and evaluation data, and to compare and analyze the impact of consumer group communication on brands in the context of differentiated group communication information authenticity perception.

## Data availability statement

The raw data supporting the conclusions of this article will be made available by the authors, without undue reservation.

## Author contributions

LW and YWu: conceptualization. YWu: methodology, software, validation, and formal analysis. YWu and YWa: data curation. LW, YWu, and YWa: writing-original draft preparation. LW: writing—review and editing and funding acquisition. All authors contributed to the article and approved the submitted version.

## Funding

This research was supported by the National Natural Science Foundation of China (71704020), the Natural Science Foundation of Heilongjiang Province (QC2017081), Postgraduate Education Research project of the National Steering Committee of Agricultural Graduate Education (2021-NYYB-04) and Scholars Plan of Northeast Agricultural University (19QC34).

## Conflict of interest

The authors declare that the research was conducted in the absence of any commercial or financial relationships that could be construed as a potential conflict of interest.

## Publisher’s note

All claims expressed in this article are solely those of the authors and do not necessarily represent those of their affiliated organizations, or those of the publisher, the editors and the reviewers. Any product that may be evaluated in this article, or claim that may be made by its manufacturer, is not guaranteed or endorsed by the publisher.
